# EPHA2 mutations with oncogenic characteristics in squamous cell lung cancer and malignant pleural mesothelioma

**DOI:** 10.1038/s41389-019-0159-6

**Published:** 2019-09-04

**Authors:** Yi-Hung Carol Tan, Saumya Srivastava, Brian M. Won, Rajani Kanteti, Qudsia Arif, Aliya N. Husain, Hubert Li, Wickii T. Vigneswaran, Ka-Ming Pang, Prakash Kulkarni, Martin Sattler, Nagarajan Vaidehi, Isa Mambetsariev, Hedy L. Kindler, Deric L. Wheeler, Ravi Salgia

**Affiliations:** 1 Section of Hematology/Oncology, Department of Medicine, The University of Chicago, Chicago, IL USA; 2 Department of Medical Oncology and Experimental Therapeutics, Comprehensive Cancer Center, City of Hope, Duarte, CA USA; 3 Department of Pathology, The University of Chicago, Chicago, IL USA; 4 Department of Molecular Immunology, City of Hope, Duarte, CA USA; 5 Loyola University Health System, Maywood, IL USA; 6 Department of Medical Oncology, Dana-Farber Cancer Institute, Boston, MA USA; 7 Department of Medicine, Harvard Medical School, Boston, MA USA; 8 Department of Human Oncology, University of Wisconsin School of Medicine and Public Health, Wisconsin Institute for Medical Research, Madison, WI USA

**Keywords:** Mesothelioma, Non-small-cell lung cancer

## Abstract

Squamous cell carcinoma (SCC) and malignant pleural mesothelioma (MPM) are thoracic malignancies with very poor prognosis and limited treatment options. It is an established fact that most of the solid tumors have overexpression of EPHA2 receptor tyrosine kinase. EPHA2 is known to exhibit opposing roles towards cancer progression. It functions in inhibiting cancer survival and migration via a ligand and tyrosine kinase dependent signaling (Y772). Whereas it is known to promote tumor progression and cell migration through a ligand-independent signaling (S897). We analyzed the expression profile and mutational status of the ephrin receptor A2 (*EPHA2*) in SCC and MPM cell lines and primary patient specimens. The EPHA2 receptor was found to be either overexpressed, mutated or amplified in SCC and MPM. In particular, the *EPHA2* mutants A859D and T647M were interesting to explore, A859D Y772 dead mutant exhibited lower levels of phosphorylation at Y772 compared to T647M mutant. Molecular Dynamics simulations studies suggested that differential changes in conformation might form the structural basis for differences in the level of EPHA2 activation. Consequently, A859D mutant cells exhibited increased proliferation as well as cell migration compared to controls and T647M mutant. Kinomics analysis demonstrated that the STAT3 and PDGF pathways were upregulated whereas signaling through CBL was suppressed. Considered together, the present work has uncovered the oncogenic characteristics of *EPHA2* mutations in SSC and MPM reinstating the dynamics of different roles of *EPHA2* in cancer. This study also suggests that a combination of doxazosin and other EPHA2 inhibitors directed to inhibit the pertinent signaling components may be a novel therapeutic strategy for MPM and Non-small cell lung cancer patients who have either *EPHA2* or *CBL* alterations.

## Introduction

Squamous cell carcinoma (SCC) is the second most common histology in non-small-cell lung carcinomas (NSCLCs) accounting for 20–30% of all NSCLC cases and more often than not, presents with advanced stage disease at diagnosis.^1^ However, compared to advanced lung adenocarcinoma, for which targeted therapies are now available, especially if they present actionable mutations, there are limited treatment options for advanced SCC, both in first-line and relapsed/refractory settings. In the last few years several new drugs that include necitumumab (anti-EGFR monoclonal antibody) in combination with standard chemotherapy, and immune-checkpoint inhibitors such as nivolumab, pembrolizumab, or atezolizumab have been approved for treating SCC.^2^ Although the response has been encouraging, new and more efficacious drugs are still needed.

Malignant pleural mesothelioma (MPM) is a neoplasm that arises from the serosal surfaces of pleura, peritoneum, and pericardium. It is a rare disease with approximately one in 100,000 people being diagnosed per year in the US. This disease most often affects individuals that have been exposed to asbestos; however, genetic factors may also have a role. Median survival from the time of diagnosis is ~9.2 months. Treatment options include surgery and/or chemotherapy.^3,4^ Unfortunately, only one chemotherapeutic agent (pemetrexed) has been approved by the FDA for treatment of this disease.^5,6^ Thus, in order to make a significant impact on the therapy and overall survival for patients with MPM, newer biological mechanisms need to be identified, and implemented therapeutically.

The erythropoietin-producing hepatocellular carcinoma (Eph) receptors that represent the largest family of receptor tyrosine kinases (RTKs),^7^ are divided into EPHA and EPHB classes based on structural homology and ligand-binding affinity.^8^ A singular feature of the Eph-ephrin system is that signals are transduced by both the receptor (forward signaling) and the ligand (reverse signaling) to form an important cell communication system with critical and diverse roles in a variety of biological processes during embryonic development.

**Fig. 1:**
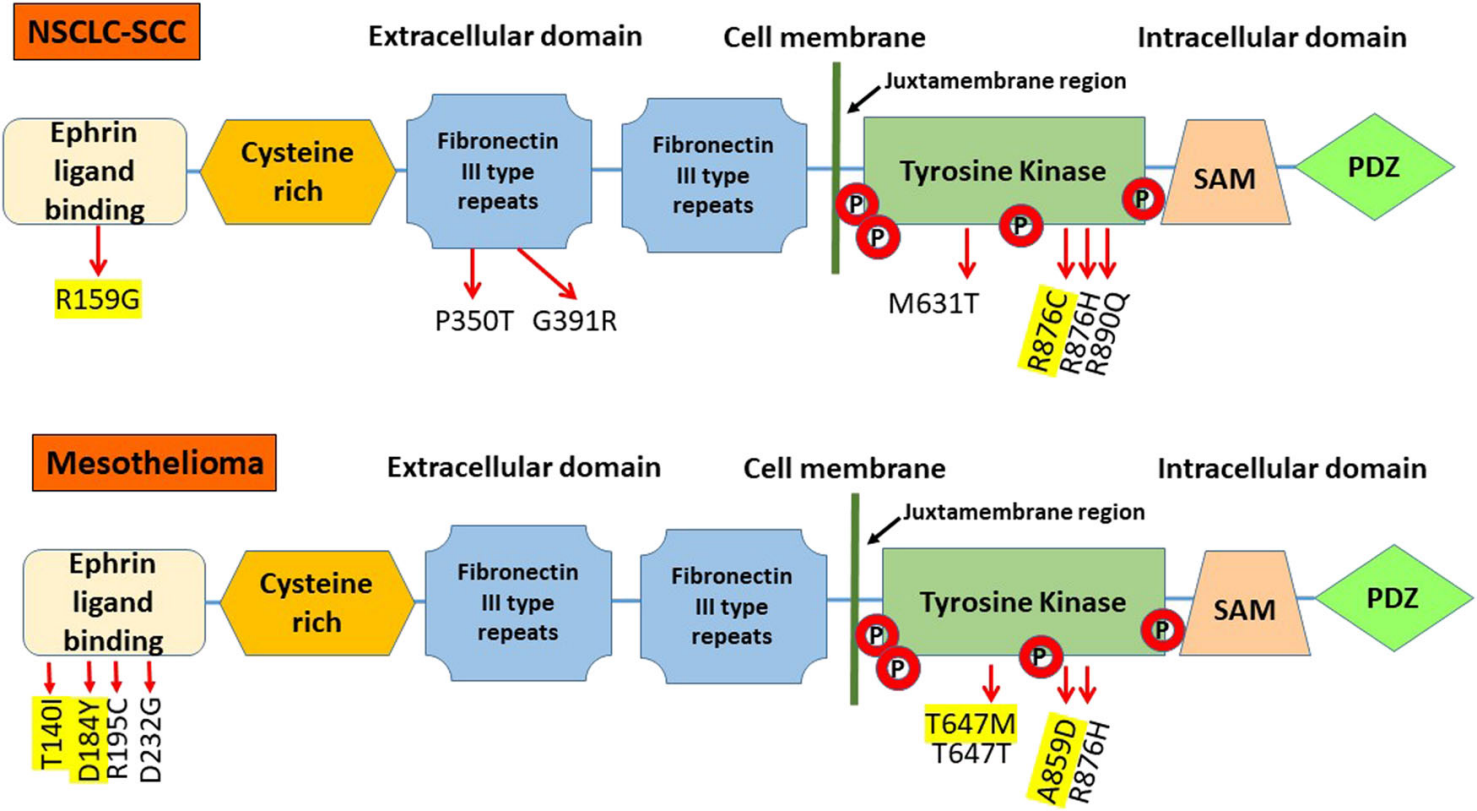
EPHA2 protein structure. EPHA2 receptor consists of the ligand-binding domain, a cysteine-rich domain, two fibronectin-III domains, a tyrosine kinase (TK) domain, a sterile alpha motif (SAM), and a PDZ-binding motif. Novel mutations (yellow) and known SNPs were found in ligand-binding domain and TK domain by us recently

EPHA2 is a 130 kDa protein consisting of 976 amino acids. The *EPHA2* gene is located on chromosome 1p and contains 17 exons spanning 31 kilobases. The extracellular region of the EPHA2 receptor consists of the ligand-binding domain, a cysteine-rich domain and two fibronectin-III domains (Fig. 1). The cytoplasmic region consists of a tyrosine kinase (TK) domain and a sterile alpha motif (SAM). The kinase domain and juxta membrane region contain multiple tyrosine residues. The two key sites here are the Y772, once activated is known to inhibit cancer cell survival and migration and S897, which aids in cancer progression.^9–12^ Phosphorylation of these tyrosine residues creates docking sites for signaling proteins containing SH2/SH3 domains such as Fyn, Src, Nck and Crk, RasGAP, LMW-PTP, PI3-kinase and the adapter proteins Grb2, Grb10, and SLAP. A number of these proteins regulate depolymerization of the actin cytoskeleton^13^ while others modulate cell adhesion.^14^ Several S/T/Y residues are phosphorylated intracellularly with important functions in vascular assembly, angiogenesis, and cell migration.^15^

Previously, it was shown that EPHA2 is expressed in lung cancer and that it is also a prognosticator of the disease.^12^ Furthermore, our previous studies on EPHB4 in NSCLC had shown that when overexpressed, EPHA2 induced the resistance to EPHB4 inhibitors.^16^ In this study, we investigated the expression, activation, and genetic alterations related to *EPHA2* in SCC and MPM as well as the potential therapeutic role of modulating EPHA2 in both diseases. Based on where the mutation occurs, *EPHA2* mutants behave differently since the mutations differentially affect EPHA2 phosphorylation and downstream signaling. We also observed inhibition with doxazosin^17^ had an additive effect with cytotoxic chemotherapies such as cisplatin.

## Results

### *EPHA2* alteration in SCC and MPM tissues and cell lines

**Fig. 2:**
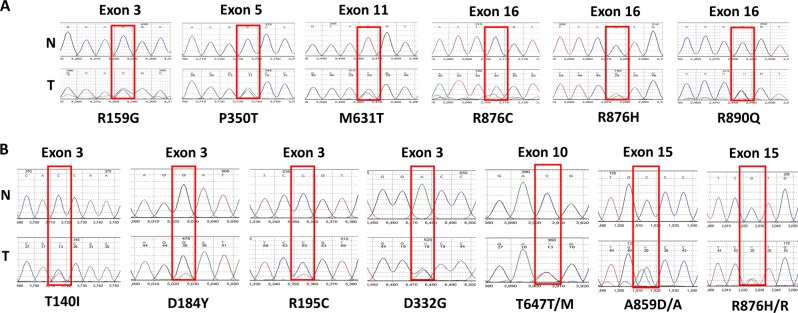
*EPHA2* gene sequencing chromatograms. Representative sequencing chromatograms of the mutation region in normal (**N**) and tumor (**T**) samples. Boxes indicate the heterozygous mutation in the tumor sample. **a** Novel mutation (R159G and R876C) and known single-nucleotide polymorphism (SNPs) (P350T, G391R, M631T, R876H, and R890Q) were detected in SSC tumor tissue. **b** Novel mutation (T140I, D184Y, T647M, and A859D) and known SNPs (R195C, D232G, T647T, and R876H) were detected in MPM tumor tissue. R159G, T140I, D184Y, R195C, and D232G were found in exon 3, P350T was found in exon 5, T647T/M were found in exon 10, M631T was found in exon 11, A859D/A and R876H/R were found in exon 15., and R876C, R876H, and R890Q were found in exon 16

**Fig. 3:**
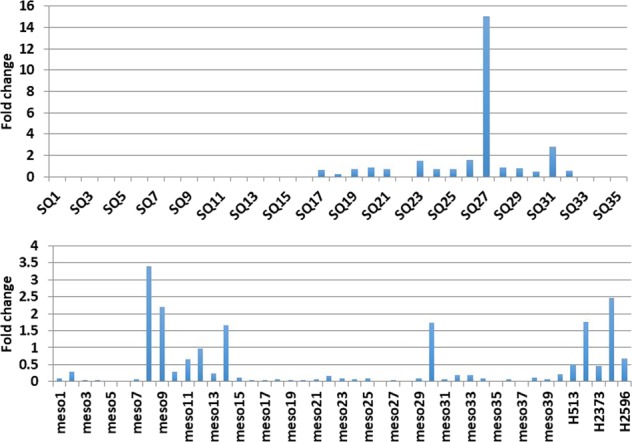
*EPHA2* gene amplification in SSC and MPM samples. Thirty five SCC, 39 MPM tumor samples, and six cell lines have been used to determine *EPHA2* gene amplification. Fold change relative to reference gene LINE-1

First, we interrogated SCC and MPM tumor samples and cell lines for genetic alterations in *EPHA2*. Genomic DNA from 35 SCC, 39 MPM patient tumors, and six MPM cell lines (H28, H513, H2052, H2373, H2461, and H2596) was used for mutation and amplification analysis. Twenty-eight percent (11/39) of MPM patient samples had *EPHA2* mutations (Fig. 2) but none were observed in SCC except for the mutant R876H. Approximately 10% (4/39) of MPM patient samples and 33% (2/6) of MPM cell lines had *EPHA2* gene amplification (Fig. 3).

### EPHA2 protein expression in MPM cell lines and in MPM and SCC tumor tissues

**Fig. 4:**
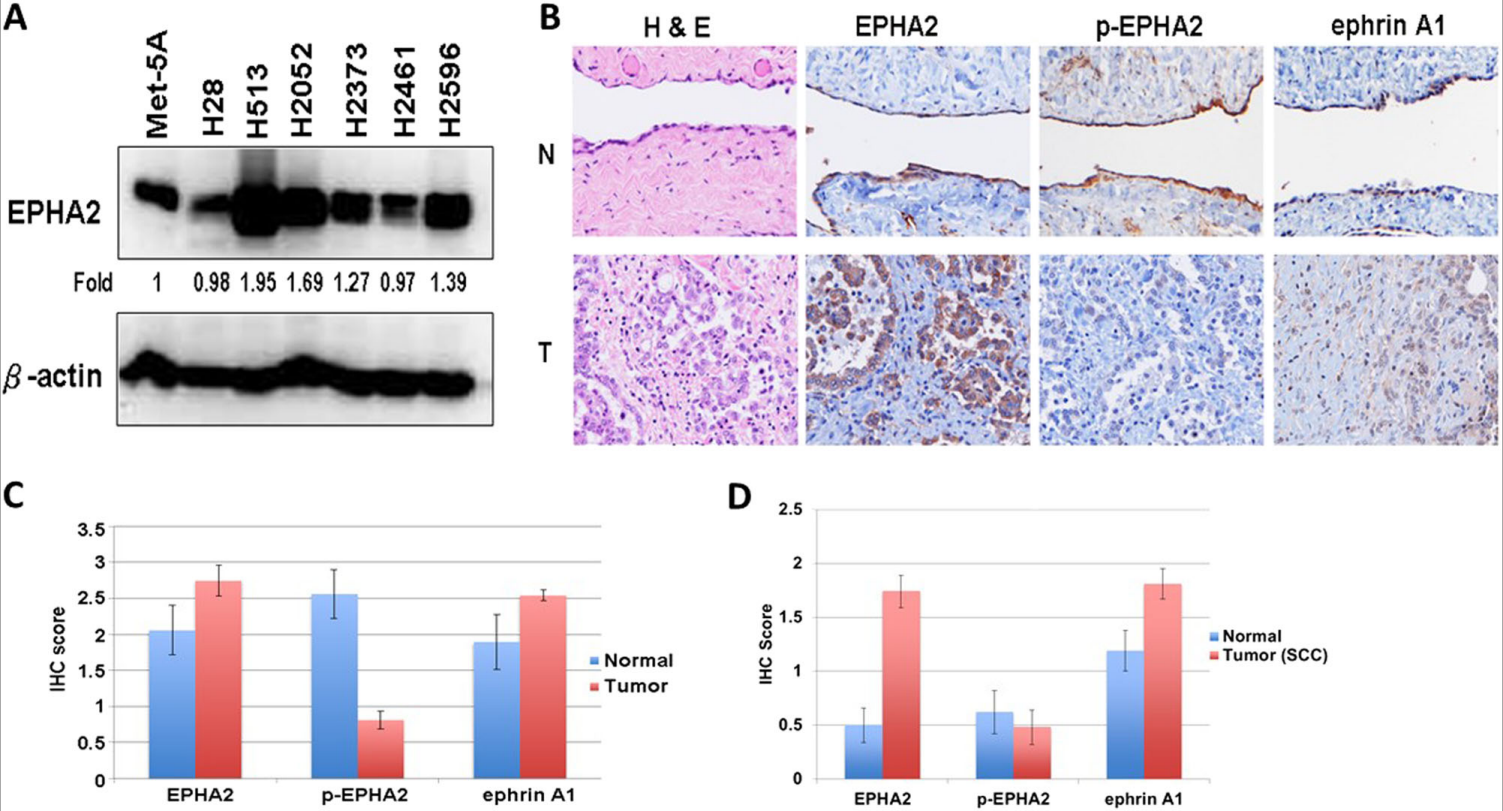
EPHA2 expression in MPM cell lines and in MPM and SCC tumor tissues. **a** Lysates of six MPM cell lines and the Met-5A, a mesothelial control cell line, were immunoblotted with EPHA2 antibody. **b** Immunohistochemistry representative pictures of 65 MPM tumor and nine normal mesothelium samples were used in MPM TMA. **c** Protein expression quantity of 65 MPM tumor and nine normal mesothelium samples were used in MPM TMA. **d** Protein expression quantity of 48 NSCLC SQ and 24 adjacent normal samples were used in SSC TMA. H&E, EPHA2, phospho-(p-)EPHA2, and ephrin A1 were stained and scored. N: normal, T: Tumor

Next, we evaluated EPHA2 protein expression in multiple MPM cell lines. Total cell lysates from six MPM cell lines (H28, H513, H2052, H2373, H2461, and H2596) and the normal mesothelial cell line Met-5A were used to determine protein expression by immunoblotting. Compared with Met-5A, the H28 and H2461 MPM cell lines showed low expression of EPHA2 while the H513, H2052, H2373, and H2596 MPM cell lines had high expression of EPHA2. In all, EPHA2 was overexpressed in 66.7% (4/6) of the MPM cell lines (Fig. 4a).

EPHA2 expression was analyzed in tumor specimens by immunohistochemistry (IHC) utilizing a tissue microarray (TMA) (Fig. 4b). The overall IHC scores for 65 MPM tumor samples and nine normal mesothelium samples of EPHA2 were 2.74 and 2.06, respectively, demonstrating that EPHA2 is overexpressed in MPM. We further determined the expression of phospho-EPHA2 and the ligand ephrin A1 (EFNA1) in the same TMA. The results showed that, in comparison with normal tissue, phospho-EPHA2 expression was low while EFNA1 was overexpressed (Fig. 4c). The overall IHC scores for the 48 SCC tumor samples and 44 adjacent normal samples for EPHA2 were 1.74 and 0.5, respectively (Fig. 4d). These results showed that, in comparison with normal tissue, phospho-EPHA2 expression is low while EFNA1 is high in MPM tumor samples.

### Effect of EPHA2 expression on cell proliferation and migration

**Fig. 5:**
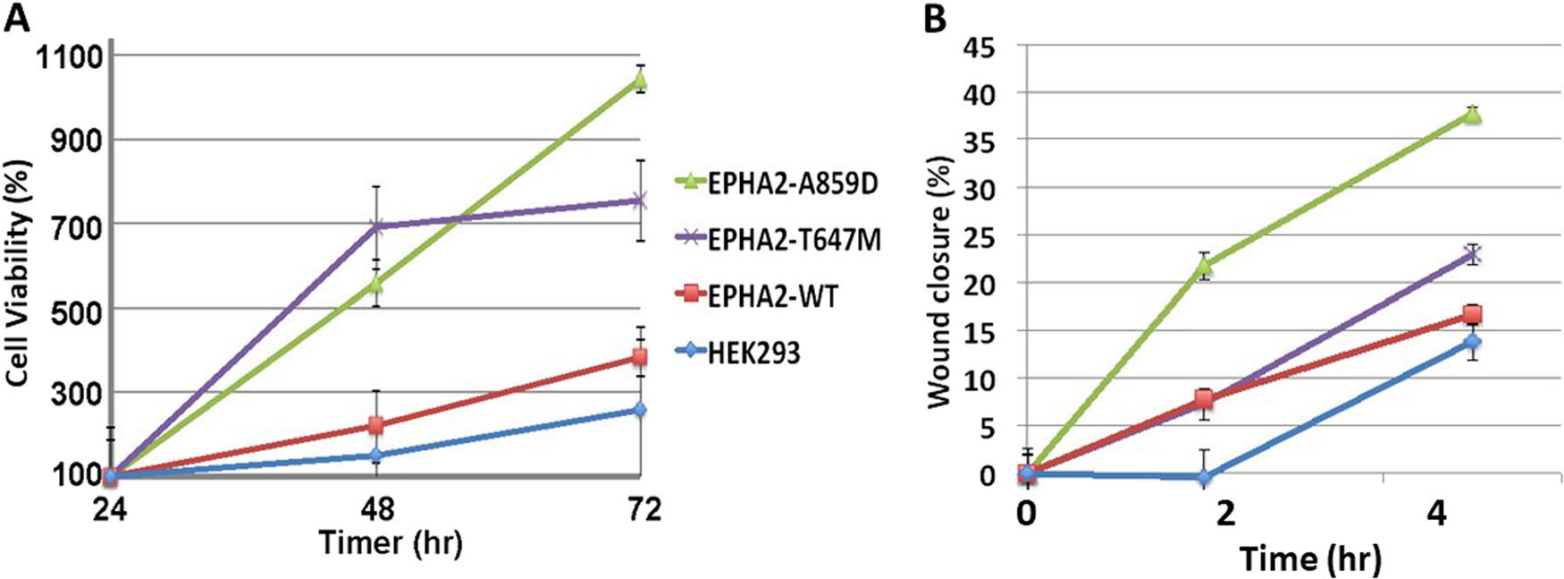
Cell viability and migration of HEK293 *EPHA2* isogenic cells. **a** Cell viability was measured every 24 h over 72 h. **b** Cell migration was measured by wound healing assay at 0, 2, and 5 h

Having established that EPHA2 is overexpressed, we next determined the effect of overexpression on cellular proliferation and migration. As shown in Fig. 5a, in HEK293 *EPHA2* isogenic cells, cell proliferation was increased in comparison with HEK239 cells that had low EPHA2 expression. Furthermore, the cells with *EPHA2* A859D and T647M mutations significantly increased proliferation compared to cells with wild-type (WT) *EPHA2* (Fig. 5a). *EPHA2* A859D mutant cells performed better than T647M cells. A wound healing assay was used to determine cell migration of HEK293 *EPHA2* isogenic cells expressing WT and mutant (Mt) *EPHA2*. Wound healing was monitored at 2 and 5 h and in both cases, cell migration was significantly increased in *EPHA2* A859D mutant in comparison with T647M mutant and HEK239 cells (Fig. 5b).

### Inhibition of cell proliferation in *EPHA2* isogenic cell lines of SCC and MPM with chemo drugs and small molecules

**Fig. 6:**
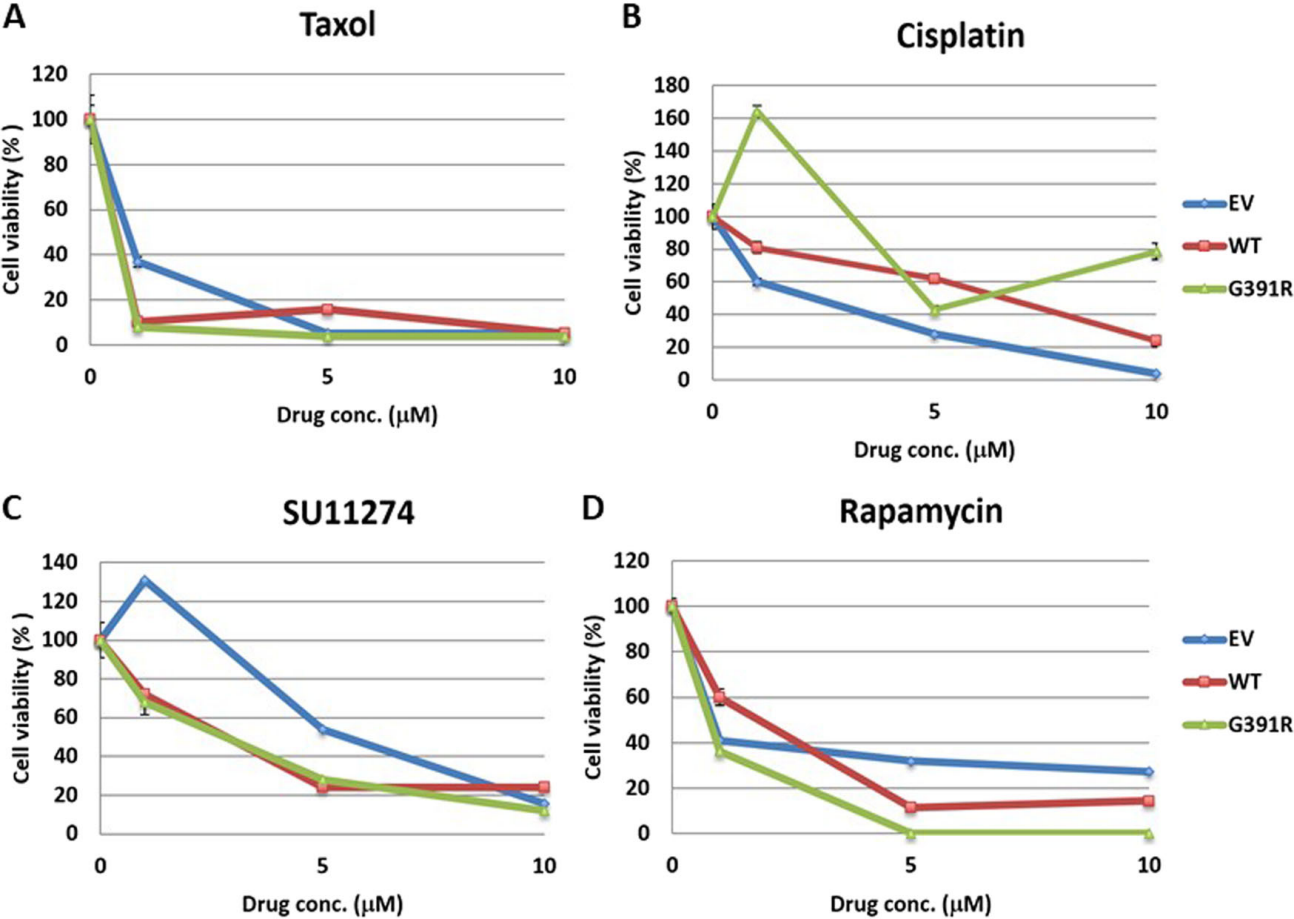
Cell cytotoxicity of SCC *EPHA2* isogenic cells with chemo drugs and small molecular inhibition. BEAS2B *EPHA2* isogenic cells were used to treat **a** Taxol, **b** cisplatin, **c** SU11274, and **d** rapamycin. Mutation G391R cells showed resistant to cisplatin inhibition but sensitive to MET inhibitor SU11274 and mTOR inhibitor Rapamycin. EV: empty vector

**Fig. 7:**
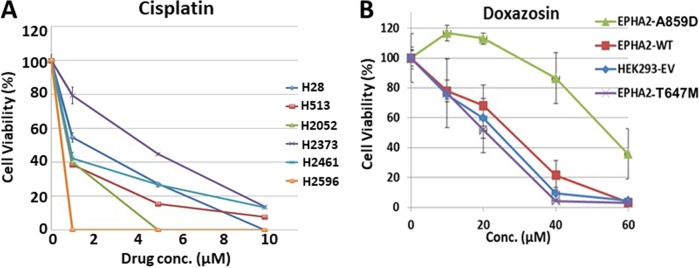
Cell cytotoxicity of HEK293 *EPHA2* isogenic cells. **a** MPM cell lines H28, H513, H2052, H2373, H2461, and H2596 were treated with cisplatin with 1, 5, and 10 μM for 48 h. **b** HEK293 *EPHA2* isogenic cells treatment with doxazosin for 48 h

To discern the effect of chemotherapy and pharmacological inhibition of EPHA2, BEAS2B *EPHA2* isogenic cells with WT and Mt receptor (G391R) were treated with the chemo drugs taxol and cisplatin and small molecule inhibitors including the MET inhibitor SU11274 and the mTOR inhibitor, Rapamycin. *EPHA2* Mt cells showed higher sensitivity to inhibition with small molecules than traditional chemo drugs compared to *EPHA2* WT cells (Fig. 6a–d). HEK293 *EPHA2* isogenic cell lines and MPM cell lines were also used to determine the effectiveness of cisplatin. MPM cell lines (H513, H2052, and H2596) that overexpressed EPHA2 showed sensitivity to cisplatin inhibition (Fig. 7a). In contrast, MPM cell lines with low EPHA2 expression (H28 and H2461) showed resistance to cisplatin treatment (Fig. 7a). In addition, HEK293 *EPHA2* isogenic cells showed inhibition with small molecule inhibitor, doxazosin. In contrast, treating HEK293 *EPHA2*-A859D Mt cells with doxazosin showed significant resistance to doxazosin (Fig. 7b). This resistance was not observed in T647M mutant, *EPHA2* WT, or empty vector control.

### *EPHA2* mutations affect multiple phosphorylation of RTKs

To elucidate the mechanism, we next determined the phospho-kinome of *EPHA2* A859D and T647M mutants with or without doxazosin treatment. The analysis showed that tyrosine kinase activity responsible for phosphorylating most peptides of EPHA2 Mts was downregulated compared to empty vector control. After treatment with doxazosin, wild-type *EPHA2* group and T647 mutant showed significant upregulation (*p* < 0.05) than *EPHA2* A859D Mts group. A volcano plot showing the changes of each group compared to empty vector group is presented in Supplementary Fig. 1.

To understand the network of affected peptides, a signal transduction network with significantly down- and upregulated peptides was predicted and created by Ingenuity Pathway Analysis (IPA®) software tool (Qiagen, Redwood City, CA) (Supplementary Fig. 2a). The analysis revealed that Tec kinase signaling, STAT3 pathway, axonal guidance signaling, T cell receptor signaling, and PDGF signaling were the top canonical pathways (*p* < 0.001) involved in all the groups with or without doxazosin treatment. In addition, cancer, post-translational modification, and cell-to-cell signaling and interaction were the networks, which had the most affected peptides involved (Supplementary Table 1 and Fig. 2b).

### Molecular dynamics simulations

**Fig. 8:**
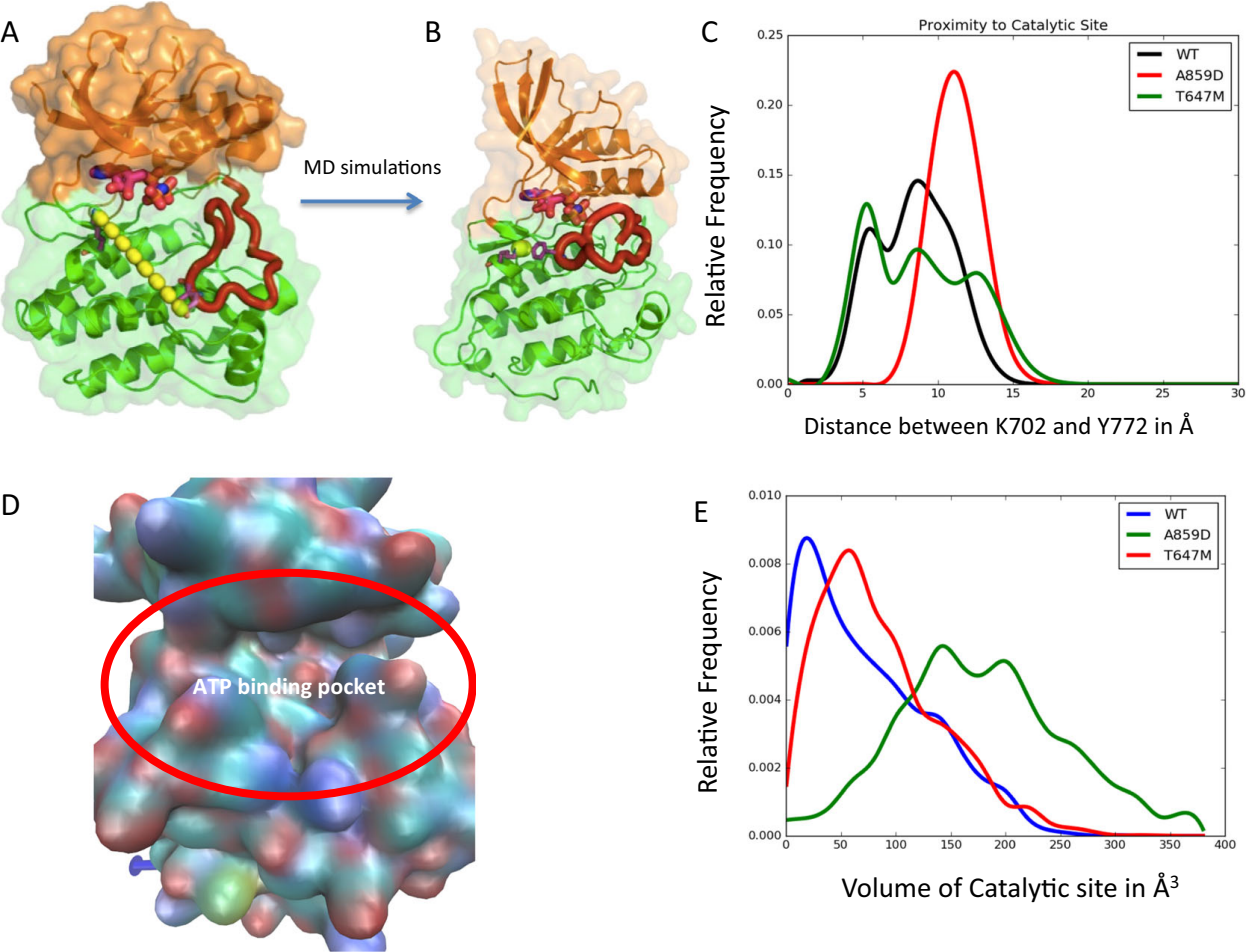
Molecular dynamics simulations. **a** The crystal structure of EphA2 in the auto-inhibited conformation. The distance between Y772 and K702 shown by dotted line is 20.3 Å, **b** The EphA2 conformation after MD simulations showing the minimum distance between Y772 and K702 which is 3.4 Å, **c** The distribution of the distance between Y772 and K702 in the wild type and mutants A859D and T647M, **d** The surface showing the ATP binding site in EphA2, and **e** The distribution of the volume in (Å^3^) of the ATP-binding site for the wild type and the two mutants calculated from the snapshots of the MD simulations

The residue Y772 in the kinase domain of EPHA2 gets auto-phosphorylated upon ligand-binding leading to the catalytic kinase activity of EPHA2. Our phosphorylation assays (Fig. 8) have shown that the mutant A859D shows low level of phosphorylation and the mutant T647M shows medium level of phosphorylation compared to the wild-type EPHA2. To rationalize this experimental finding, we performed molecular dynamics (MD) simulations on the kinase domain structure of EPHA2 starting from the crystal structure of the auto-inhibited conformation of EPHA2, in explicit water as described in the Methods section. We also performed MD simulations of the two mutations A859D and T647M under the same conditions as the wild type. Our hypotheses are two-fold: (i) the residue Y772 in EPHA2 should get close (within 4 Å) to the γ-PO_4_ group of ATP to facilitate the phosphoryl transfer to Y772. (ii) The mutations A859D and T647M could affect the dynamic proximity of the γ-PO_4_ of ATP to Y772 thereby reducing the phosphorylation of Y772. Additionally, the shape of the ATP binding site could be affected by the mutations thereby affecting the binding affinity of ATP to EPHA2. K702 is a residue that binds close to the γ-PO_4_ of ATP and hence we used this residue to locate the ATP binding site. Figure 8a shows that the distance between Y772 and K702 is 23.7 Å in the crystal structure of the auto-inhibited state of EPHA2. Figure 8b shows the minimum distance between Y772 and K702 achieved during the MD simulations. This shows that the dynamics of the wild-type EPHA2 brings the Y772 close enough to ATP to get phosphorylated. Figure 8c shows the distribution in the distance between Y772 and K702 during the MD simulations of the wild type and the two mutants. It is seen that the wild-type and T647M mutant show closer distances between Y772 and K702, the mutant A859D does not get Y772 close to K702 during the dynamics of the kinase domain. This could impair the phosphorylation of Y772 in the A859D mutant compared to the wild-type. The distance distribution in T647M is broader than that of the wild-type. We have also calculated the volume of the binding cavity of ATP as shown in Fig. 8d, and the distribution of the volume in (Å^3^) of the ATP binding site for the wild-type and the two mutants calculated from the snapshots of the MD simulations is shown in Fig. 8e. The distribution of the volume of the ATP binding site is the broadest for A859D, followed by T647M and then the wild-type. This shows that the ATP binding site is very flexible in A859D mutant that could possibly lead to faster off-rate of ATP and thus reducing the binding affinity of ATP and therefore it’s potential to phosphorylate Y772 in the mutant A859D EPHA2. These differential changes in conformation captured by the MD simulations provide a structural basis for the differences in the level of Y772 phosphorylation and explains the trend observed in the phenotypic changes.

## Discussion

**Fig. 9:**
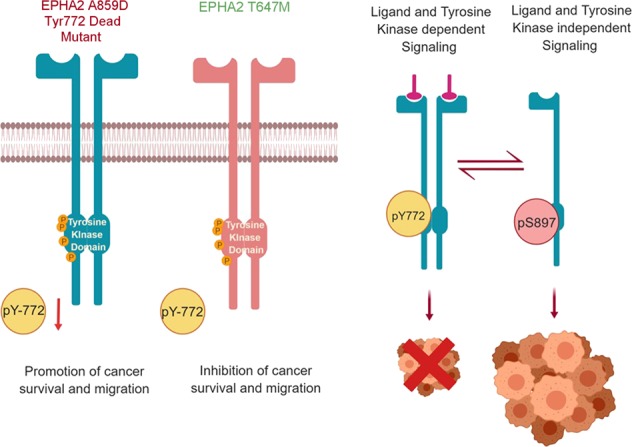
The EPHA2 mechanistic model. The model depicts how in the A859D mutant loss of Y772 phosphorylation leads to enhanced tumorigenesis in comparison to T647M mutant

In this study, employing a variety of biological, biochemical, and computational tools, we have uncovered the oncogenic vs tumor suppressor characteristics of *EPHA2* and its mutations in SSC and MPM. In addition to identifying novel mutations, the *EPHA2* gene is amplified or the protein is overexpressed in these two diseases. It is well established that, in cancer cells, EPHA2 protein is typically not phosphorylated due to the overexpression of LMW-PTP phosphatase in these cells.^18^ Thus, the decreased levels of p-EPHA2 and enhanced levels of total EPHA2 appear to result from enhanced protein stability.^19^ Nonetheless the phosphorylation status of EPHA2 leads to differential activation of signaling pathways resulting in different outcomes in terms of cancer progression. The tumorigenic signature of EPHA2 is defined by ligand-independent high levels of S897 phosphorylation and ligand- and kinase-dependent low levels of Y772 phosphorylation. Therefore, among the several novel mutations identified in this study, the mutations in the tyrosine kinase domain in particular were assayed. Mutations A859D and T647M displayed contrasting phenotypes. A859D, the Y772 dead mutant, showed increased cell proliferation and migration of MPM cells in comparison to T647M mutant and control cells. Furthermore, we also observed a stimulation of growth with low dose of cisplatin and SU11274 treatment (Fig. 6b, c). Also, *EPHA2* mutation A859D, conferred resistance to targeted therapy as well as cisplatin chemotherapy. The Y772 phosphorylation is much reduced in A859D mutant than in T647M mutant. To understand the difference seen in the phenotypes of these two mutants, we carried out computational MD simulation studies to model the dynamics of the kinase domain and probe the intramolecular interactions that lead to phosphorylation of Y772. Our analysis of the MD simulation trajectories showed that the A859D mutant impairs the phosphorylation by reducing the flexibility of the loop containing Y772 and therefore getting closer to the ATP for direct phosphoryl transfer. Additionally, the EPHA2 A859D mutant shows increased volume of the ATP binding site compared to the wild-type EPHA2, that leads to faster off-rate of ATP and lowering the affinity of ATP to this mutant. These two factors together provide atomic-level insights into the impairment of phosphorylation of Y772 in the mutant A859D. This explains why we see decreased phosphorylation at Y772 in A859D cells. The absence of the inhibiting phosphorylation in A859D mutant, leads to increased cell proliferation and migration and reduced sensitivity to chemotherapeutic or small molecule drug treatments Fig. 9.

We also found that CBL was downregulated in H2373 *EPHA2* isogenic cells (low EPHA2 expression) and upregulated after treatment with doxazosin. CBL is an E3 ubiquitin ligase and an adaptor molecule that plays an important role in NSCLC.^20^ CBL is often observed to have low expression in cancer cells especially in NSCLC. Interestingly, our study showed that low expression of CBL results in MET overexpression and increased cell proliferation and migration suggesting that patients with low CBL expression or mutation may benefit from MET inhibitor, e.g., SU11274 due to increased expression of MET.^21^ Lastly, in light of the fact that doxazosin treatment results in upregulation of CBL, a combination of doxazosin and other EPHA2 inhibitors may be a novel therapeutic strategy for MPM and NSCLC patients who have either *EPHA2* or *CBL* alterations.

## Materials and methods

### Source of sample materials

The research on human subjects in this paper utilized tissue samples from patients with cytologically or histologically documented SCC and MPM. Samples utilized were archival samples already banked by the University of Chicago Human Tissue Resource Center, Clinical Research Center, and Pathology Department. These tissues were obtained with subject consent (under IRB protocols #9571 and #13473 A) or under consent-waived protocols for deceased or de-identified patients. For all samples, clinical information (date of diagnosis, survival, asbestos exposure, etc.) was obtained from patient medical records and/or the Thoracic Oncology Research Program (TORP) database at the University of Chicago, developed by the Salgia laboratory that is freely available on the iBridge Network website (http://www.ibridgenetwork.org/uctech/salgia-thoracic-oncology-access-template).^22,23^

### EPHA2 expression

Expression of EPHA2 was assessed in tumor tissues obtained from patients with various histologies of SCC and MPM. We utilized tumor tissue microarrays (TMAs) and performed immunohistochemistry (IHC) with antibodies against EPHA2 and phospho-EPHA2 as well as the EPHA2 ligands ephrin A1, EPHA3, and EPHA3 ligand ephrin A5. EPHA3 and its ligand were used as a potential alternative Eph receptor. SSC and MPM TMAs were constructed from archival tumor tissues obtained from untreated patients, diagnosed in 2003 or later and 30 normal controls. For each patient/control, two replicate tissue samples were evaluated. Immunostained TMAs were blindly scored by pathologists utilizing bright-field microscopy. IHC score is calculated by taking into account the strength of staining intensity combined with the extent of staining area to determine a final score (0, negative; 1 + , weak; 2 + , moderate; and 3 + , strong expression). MPM cell lines: H28, H513, H2052, H2373, H2461, and H2596 were analyzed for protein expression by immunobloting as described previously.^16^ Met-5A, the normal mesothelial cell line, was used as control.

### Quantitative real-time PCR

Quantitative real-time PCR using DNA samples obtained from tumors of patients with SCC and MPM was performed to determine the gene amplification. The PCR primers used are listed in Supplementary Table 2. StepOne real-time PCR system and Power SYBR Green PCR Master Mix (Applied Biosystems) were used to perform the experiment. Relative gene copy number was calculated from the real-time PCR efficiencies, which were determined for each individual run, and the crossing point deviations of the target and reference genes in a test sample versus a control. Long interspersed element-1 (LINE-1) served as reference gene, which is a repetitive element for which copy numbers per diploid genome are similar in healthy or malignant human cells.^24^ Reactions were done in triplicate under standard thermocycling conditions (one cycle of 95 °C for 10 min, followed by 40 cycles of 95 °C for 30 s and 58 °C for 1 min), and the mean threshold cycle number was used.

### Gene mutation analysis

Exons 1–17 of *EPHA2* gene were individually amplified by PCR. The primers are listed in Supplementary Table 3. PCR conditions were as follows: 1 cycle of 95 °C for 5 min; 35 cycles of 94 °C for 30 s, 58 °C for 30 s and 72 °C for 2 min; and one cycle of 72 °C for 10 min. PCR products were treated with ExoSAP-IT (USB Corporation, Cleveland, OH, USA) and sequenced by Big-Dye Terminator Chemistry (Applied Biosystems, Foster City CA). Sequencing was performed on the forward coding strand with confirmation of *EPHA2* alterations performed by sequencing the reverse strand as well. Chromatograms were analyzed for mutations using Mutation Surveyor v2.61 software (Softgenetics, State College, PA).

### Plasmid constructs and site-directed mutagenesis

The wild-type *EPHA2* construct (pCMV6-AC-GFP–EPHA2) was purchased from OriGene (Rockville, MD). Using this parental plasmid, the TKB domain mutation T647M and A859D were created using the following primers: (Forward) 5’-GTGGCCATCAAGATGCTGAAAGCCGG-3’/ (Reverse) 5’-GCCGGCTTTCAGCATCTTGATGGCCAC-3’ and (Forward) 5’-CAGGAGCGTGACCGCCGCCCC-3’/ (Reverse) 5’-GGGGCGGCGGGTCACGCTCCTG-3’, respectively, along with their complementary primers using the QuickChange Site-Directed Mutagenesis XL kit (Stratagene, La Jolla, CA) following the manufacturer’s instructions. Point mutations were confirmed by standard DNA sequencing of both strands.

### Transfection of EPHA2 constructs

HEK293 and MPM cell lines, which expressed low EPHA2, were transfected using the Fugene HD (Roche, Nutley, NJ) reagent according to the manufacturer’s instructions. We utilized HEK293 cells as a high-throughput model for determining the efficacy of the plasmid and transfection efficiency. Eight microgram of plasmid DNA, containing either no insert (empty vector), wild-type (WT) *EPHA2*, A859D *EPHA2*, or T647M *EPHA2* was used for transfection in a six-well culture plate. Transfection efficiency was detected by observing GFP expression under microscope after 24 h transfection.

### *EPHA2* knockdown

*EPHA2* knockdown was performed by transfecting cells with *EPHA2* shRNA (OriGene, Rockville, MD) as per manufacturer’s instructions. Briefly, 1 × 10^5^ cells/well of H513 or MPM cell lines with high EPHA2 expression were seeded in six-well plates and transfected the following day with *EPHA2* shRNA constructs. To generate stable *EPHA2* knockdown cell lines, cells were selected for 1–2 weeks with 1 μg/ml puromycin. EPHA2 expression levels were determined in whole-cell lysates by immunoblotting using anti-EPHA2 antibody (Santa Cruz Biotechnologies, Santa Cruz, CA).

### Cell viability assay and drug treatment

*EPHA2* isogenic cells were transfected with *EPHA2* WT and mutants (Mts) as described above. forty-eight hour after transfection, cells were harvested and re-seeded at 5 × 10^4^ cells/well in a 24-well culture plate. Cell viability was monitored 24, 48, and 72 h and determined using Calcein-AM exclusion. After seeding the cells for 24 h, cells were treated with cisplatin, taxol, rapamycin, SU11274, or doxazosin and monitored 24, 48, and 72 h and determined using Calcein-AM exclusion.

### Wound healing assay

*EPHA2* isogenic cells were transfected with *EPHA2* WT and Mts as described above. Cells were seeded in six-well plates and cultured for 48 h until 100% confluent. After resting the cells for 12 h, a straight scratch was made across the cell layer using a 1 ml pipette tip. The cells were then gently washed with 1X PBS to remove cellular debris and the media was replaced. Photographs of the wound region were taken 0, 2, and 5 h had passed. The images were analyzed using the TScratch software (Computational Science and Engineering Laboratory, ETH Zurich, Switzerland).

### Immunoblotting

Cells were collected at 48 h after transfection and washed twice in 1X PBS, then lysed with ice-cold lysis buffer (0.5 M Tris-HCl with pH 7.4, 1.5 M NaCl, 2.5% deoxycholic acid, 10 mM EDTA, 10% NP-40, 0.5 mM DTT, 1 mM phenylmethylsulfonyl fluoride, 5 µg/mL leupeptin, and 10 µg/mL aprotinin) for 5 min. The lysate was centrifuged at 13,000 rpm for 20 min at 4 °C, and protein content of the supernatant was measured. Total cell lysates (50 µg/well) were separated by SDS-PAGE electrophoresis and the gels transferred onto nitrocellulose membranes (Whatman, Piscataway, NJ). Membranes were blocked with 5% non-fat dry milk in PBST (1X PBS, 0.1% Tween-20) for 1 h at room temperature and incubated with the appropriate primary antibody at 4 °C overnight. Membranes were then washed three times with PBST and probed with the appropriate HRP-conjugated secondary antibodies for 1 h at room temperature. The membranes were again washed three times in PBST and bands were visualized using western blot chemiluminescence reagent (BioRad, Valencia, CA) on a Chemidoc Gel documentation system (BioRad). The following antibodies specific for EPHA2, phospho (p)-EPHA2 (S897), p-EGFR (Y1125), p-EPHA4, p-EPHA7 (Y60), and p-STAT1 (Y701) were used.

### PamGene technology and analysis

The kinase activity of H2373 *EPHA2* isogenic cells with and without doxazosin was monitored using PamChip® Tyrosine Kinase Array. Briefly, cells were treated with doxazosin, harvested, and lysed in Mammalian Extraction Buffer (M-PER, Pierce) containing phosphatase and protease inhibitors (HALT, Pierce). Following lysis, 5 μl of the lysis solution was pipetted into a reaction mixture composed of 1X ABL buffer (New England Biolabs), 0.1% Bovine Serum Albumin, 100 μM ATP, 20 μg/ml phosphotyrosine antibody in a total volume of 40 μl. PamChip arrays were blocked with 0.2% BSA prior to loading the samples. After loading the reaction mixtures onto the PamChip arrays real-time data for the kinase activity were obtained by measuring fluorescence of the bound anti-phosphotyrosine antibody after each of the five cycles. Image quantification and data processing were conducted with dedicated PamGene software Evolve and BioNavigator (PamGene). The peptides that were significantly differentially affected peptides and signaling pathways were analyzed using Ingenuity IPA (Redwood City, CA).

### Computational methods

#### Modeling of the EPHA2 kinase domain

The three-dimensional structures of two different conformations of the EPHA2 kinase domain were taken from the protein data bank (PDB) (http://www.rcsb.org). The PDB codes for these two structures are: 1MQB and 5EK7.^25^ These two structures both represent the “inactive” conformations of the kinase domain. The “inactive” conformation sequesters the residue Y772 (the phosphorylated residue) away from the ATP binding site. This renders the kinase inactive. The two crystal structures were selected because together both these structures compensated for the missing activation loop structures in each other. The wild-type sequence of the kinase domain of EPHA2 was obtained from UniProt database and the missing residues numbered 586–605, 634–638, and 887–893 were modeled with MODELLER using the two listed structures as templates. A total of 50 homology models for the missing loops were generated and the models were evaluated by DOPE score and favorable number of contacts formed between the residues along the C-terminus that was added and the main body of the protein. Mutant structures were then generated using the Schrodinger *Maestro* program from Schrodinger Inc. to create each SNP.^26^ The side chain conformations of the mutation residue and the neighboring amino acids within 5 Å were optimized using *Prime* module (also from Schrodinger Inc.).^27^ The side chain optimized model was then minimized in energy.

#### Molecular Dynamics (MD) Simulation setup

MD Simulations were run using GROMACS version 5.0.5. The GROMOS96 53a6 force field was selected and sufficient Cl^-^ ions were added to neutralize each system.^28–32^ Each model was solvated in a dodecahedron using SPC/E water molecules with 10 Å to the boundary edges. Each system was then minimized for a maximum of 500 steps by the steepest decent algorithm until the maximum force was below 1000 kJ/mol/nm. NVT equilibration was carried out on each minimized structure for 500 picoseconds to 310 K.^33^ NPT equilibration was then carried out for 500 picoseconds using the Parrinello-Rahman barostat to equilibrate the system at 1 atmosphere-pressure.^34^ One replicate used the initial velocities generated during equilibration and two other replicates were initiated using random velocities. The three replicates were run for a total of 350 nanoseconds each for a collective simulation time of 1.05μs.

#### Computational analysis

Trajectories were concatenated processed using VMD.^35^ Distances were calculated in Å, between the NZ atom of K702 and the OH atom of Y772. Individual representations of EPHA2 were visualized using PyMOL.^36^ The volume of the catalytic site was calculated by selecting one frame every 200 picosecond POVME 2.0.^37,38^ The script was modified to calculate the volume at the centroid of the CA atoms of A644, Q656, D659, A699, and G776. The graphs of distance and volume were created using Matplotlib, structural representations were generated using PyMOL, and binding volume representations were generated using VMD.^36,39^

## Supplementary information

Supplementary tables

Supplemental fig legends

Supplementary Fig 1. PamGene analysis. H2373 EPHA2 isogenic cells were treated with/ without doxazosin. The volcano plot showed the changes of wild-type, A859D, and T647M compared to H2373 empty vector control. Each spot represents a test on one of the 143 substrate peptides on PamChip®. The vertical axis shows significance. Significant peptides with p< 0.01 (significance > 2, the red spots in the plot)

Supplementary Fig 2. Networks of affected peptides. (A) Networks of affected peptides were predicted by IPA. Cancer, post-translational modification, and cell-to-cell signaling and interaction were the networks which had the most affected peptides involved. (B) Immunoblotting showed effected RTKs by EPHA2 mutations and Doxazosin treatment. The square and the arrow indicated the changes reflected from (A). M: protein marker, EV: empty vector control, WT: wild-type EPHA2
