# Supplementary material for: EPHA2 mutations with oncogenic characteristics in squamous cell lung cancer and malignant pleural mesothelioma
Source: Oncogenesis. 2019 Sep 4;8(9):49. doi: 10.1038/s41389-019-0159-6 (PMC6726628; doi:10.1038/s41389-019-0159-6)
Supplement: Supplementary file 1 — Supplementary tables [file 41389_2019_159_MOESM1_ESM.docx]

**Supplementary Table 1. Summary of PamGene analysis.**

|  | **Isogenic no treatment** | | | **Doxazosin** | | |
| --- | --- | --- | --- | --- | --- | --- |
| **Potential upstream regulator** | β-estradiol,  GSK690693 (AKT inhibitor. Inhibited WT, A859D, and T647M),  ESR1,  CBL,  APP | | | CBL,  BMS-754807,  β-estradiol,  tretinion,  GSK690693 (activated WT and inhibited A859D) | | |
| **Top Canonical pathways** | Tec kinase signaling, STAT3 pathway, Axonal guidance signaling, T cell receptor signaling, PDGF signaling | | | Tec kinase signaling STAT3 pathway Axonal guidance signaling T cell receptor signaling PDGF signaling | | |
| **Up-regulated genes** | **WT** | **A859D** | **T647M** | **WT** | **A859D** | **T647M** |
|  | ANXA1, ANXA2, PLCG1, PDPK1, EPHA4, CTTN, TNNT1, STAT1, EPHA7, RBL2 | RET, EFS, STAT1, STAT3 | RET (not significant), EFS, STAT1 | CBL, RET, ANXA2, TNNT1, PDPK1, EPHA7, LAT, EGFR, CTTN, EPHA1 | CBL, RET, ANXA1, ANXA2, TNNT1, STAT1 | CBL, RET, ANXA1, ANXA2, EFS, CD247, PLCG1, TNNT1, CTTN, EPHB1 |
| **Down-regulated genes** | **WT** | **A859D** | **T647M** | **WT** | **A859D** | **T647M** |
|  | CBL, RET, DDR1, EPHB1, EGFR, MBP, STAT3, CRK, CDK7, DCX, C1R | CBL (not significant), LAT, TNNT1, EPHA1, CTTN, FRK, PLCG1, PDPK1, EPHA7, CD247, EGFR | CBL, TNNT1, EPHA1, EPHA7, LAT, FRK, EPHB1, EGFR, CTTN, PDPK1, ERBB2 | MBP, FGFR3, EPHA4, NTRK2, STAT4, MST1R, CRK, RAF1, NCF1, EPHB4 | EGFR, DDR1, PDPK1, NTRK2, LAT, MST1R, CTTN, INSR, CD79A, PLCG1 | EGFR, DDR1, CDK7, FGFR3, NTRK2, EPHB4, MST1R, TEC, NTRK1, STAT1 |

**Supplemental Table 2. EPHA2 QPCR primers**

|  | **Forward (5’->3’)** | **Reverse (5’->3’)** |
| --- | --- | --- |
| **1** | **GTGTACCGAGGAGAGGCTGA** | **CCGACTCGGCATAGTAGAGG** |
| **2** | **ACTGCCAGCATCTCTCGTTT** | **ATGCTCAACATCACCGATCA** |
| **LINE-1** | **AAAGCCGCTCAACTACATGG** | **TGCTTTGAATGCGTCCCAGAG** |

**Supplemental Table 3. EPHA2 PCR amplification primers for mutation analysis**

| **Exon** | **Forward (5’->3’)** | **Reverse (5’->3’)** |
| --- | --- | --- |
| **1** | **GGACCGAGAGCGAGAAGC** | **CGACACCAGGTAGGTTCCAA** |
| **2** | **TTTCCTGCCATGGTCTGACT** | **GGCTGAGCTGCTGAATTGAA** |
| **3** | **GGAGAGCACGAACTGGAAAG** | **AACCCAGAACCGTCACTCAC** |
| **4, 5** | **GGGTGGAAGCAGATTGAACT** | **GTCCTCCTTAAGCCCCACCT** |
| **6, 7** | **TCTTACCCACTTCCCCCACT** | **TCAATTGCTTGGTTCTGGGC** |
| **8** | **AATTTGTGGGGGAACTCCCTTG** | **TGAGGAAATGGAGGTTCCTG** |
| **9** | **TCACTGACCTCCCTGCATTT** | **ATCTTCAGAGACTTGGACCAGGCT** |
| **10, 11** | **TTCTCCTCTGACTCCAGAGCA** | **ACAGACAGAGCCCCTGCTAAGT** |
| **12** | **ACCTTCCCCCATATCTGTGC** | **CCCCTACAACCCACATCCTT** |
| **13, 14** | **GGATGCCTGGCCATTGAG** | **TTGAGATGAGTAAAGGGCTTGAG** |
| **15** | **TGCAGAGGGTTTCAGTGGCTTT** | **GTGGTTCAAGAGTCTGCAGAAG** |
| **16** | **CACCTGCAGTGCTTCCTGT** | **GGAAAGGGGCCTGACTTAC** |
| **17** | **CAGCCCCTAACTCTCCCTCT** | **AGGGAGGCCACTCTGTTTCT** |
