# Supplementary material for: EPHA2 mutations with oncogenic characteristics in squamous cell lung cancer and malignant pleural mesothelioma
Source: Oncogenesis. 2019 Sep 4;8(9):49. doi: 10.1038/s41389-019-0159-6 (PMC6726628; doi:10.1038/s41389-019-0159-6)
Supplement: Supplementary file 2 — Supplemental fig legends [file 41389_2019_159_MOESM2_ESM.docx]

**Supplemental Figure Legends**

**Supplemental Fig. 1: PamGene analysis.** H2373 *EPHA2* isogenic cells were treated with/ without doxazosin. The volcano plot showed the changes of wild-type, A859D, and T647M compared to H2373 empty vector control. Each spot represents a test on one of the 143 substrate peptides on PamChip®. The vertical axis shows significance. Significant peptides with p< 0.01 (significance > 2, the red spots in the plot).

**Supplemental. Fig.2: Networks of affected peptides. (A)** Networks of affected peptides were predicted by IPA. Cancer, post-translational modification, and cell-to-cell signaling and interaction were the networks which had the most affected peptides involved. **(B)** Immunoblotting showed effected RTKs by EPHA2 mutations and Doxazosin treatment. The square and the arrow indicated the changes reflected from (A). M: protein marker, EV: empty vector control, WT: wild-type EPHA2.
