# Supplementary figures and images for: EPHA2 mutations with oncogenic characteristics in squamous cell lung cancer and malignant pleural mesothelioma
Source: Oncogenesis. 2019 Sep 4;8(9):49. doi: 10.1038/s41389-019-0159-6 (PMC6726628; doi:10.1038/s41389-019-0159-6)

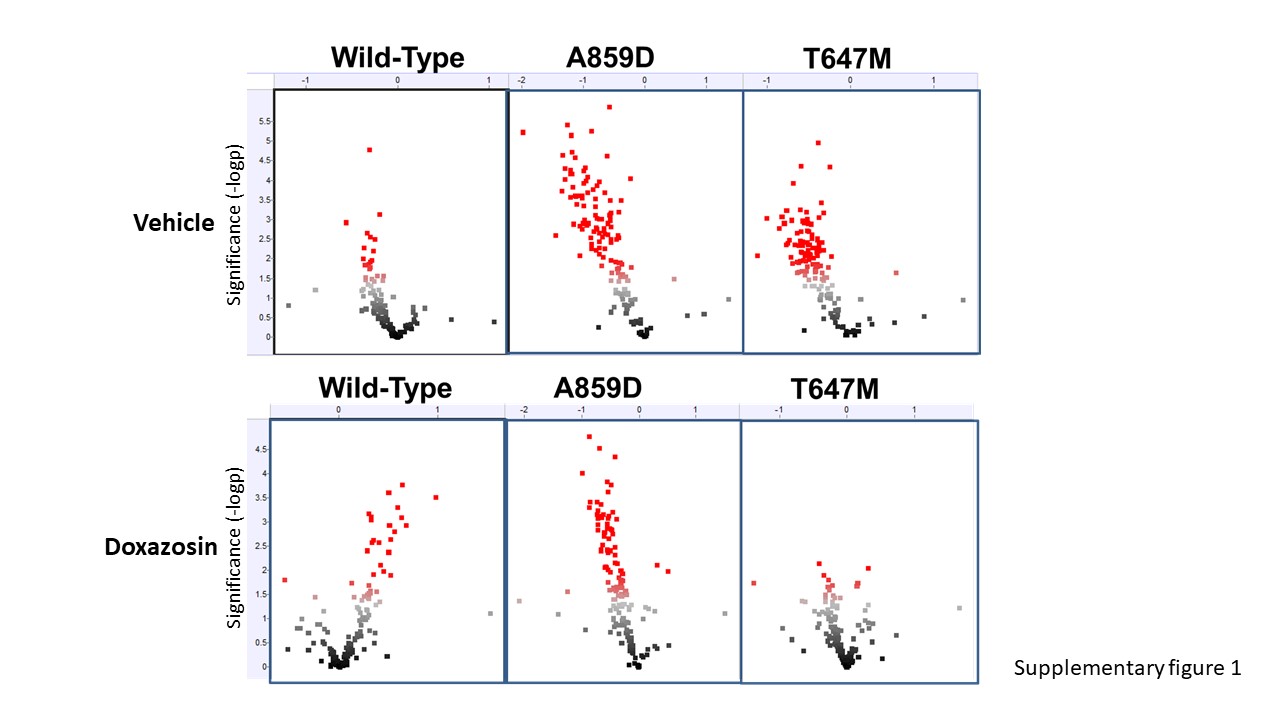

Supplement: Supplementary file 3 — Supplementary Fig 1. PamGene analysis. H2373 EPHA2 isogenic cells were treated with/ without doxazosin. The volcano plot showed the changes of wild-type, A859D, and T647M compared to H2373 empty vector control. Each spot represents a test on one of the 143 substrate peptides on PamChip®. The vertical axis shows significance. Significant peptides with p< 0.01 (significance > 2, the red spots in the plot) [file 41389_2019_159_MOESM3_ESM.jpg]

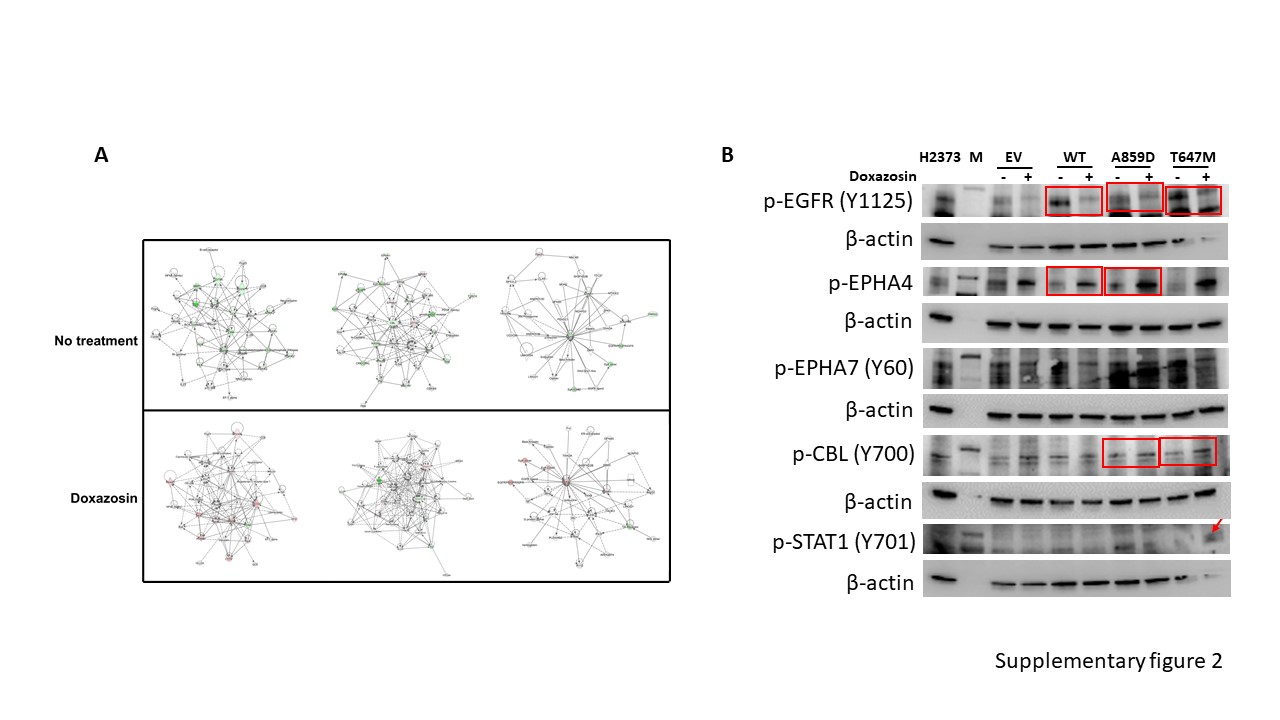

Supplement: Supplementary file 4 — Supplementary Fig 2. Networks of affected peptides. (A) Networks of affected peptides were predicted by IPA. Cancer, post-translational modification, and cell-to-cell signaling and interaction were the networks which had the most affected peptides involved. (B) Immunoblotting showed effected RTKs by EPHA2 mutations and Doxazosin treatment. The square and the arrow indicated the changes reflected from (A). M: protein marker, EV: empty vector control, WT: wild-type EPHA2 [file 41389_2019_159_MOESM4_ESM.jpg]
